# Exploring the initial experience of hospitalisation to an acute psychiatric ward

**DOI:** 10.1371/journal.pone.0203457

**Published:** 2018-09-04

**Authors:** Agnes Chevalier, Eleni Ntala, Catherine Fung, Stefan Priebe, Victoria J. Bird

**Affiliations:** 1 Unit for Social and Community Psychiatry, Blizard Institute, Queen Mary University of London, London, United Kingdom; 2 East London NHS Foundation Trust, London, United Kingdom; Newcastle University, UNITED KINGDOM

## Abstract

**Background:**

Patient-reported satisfaction with inpatient psychiatric services, within the first few days of admission, is related to positive future outcomes. Despite its predictive value, little is known about this initial experience and what underlies these appraisals. The aim of this study was to qualitatively explore the initial experience of being admitted to an inpatient psychiatric ward.

**Methods:**

Semi-structured interviews were conducted with 61 recently admitted patients across five psychiatric hospitals in London, England. Participants were purposively sampled to ensure a mix of experiences including people with high and low satisfaction scores as measured by the Client Assessment of Treatment. Thematic analysis was used to identify, analyse and report patterns within the data, with content analysis applied to determine whether certain themes were more common to either negative or positive appraisals.

**Results:**

Four broad themes were evident 1) ‘Best place for me right now?’ 2) ‘Different from out in society’ 3) ‘Moving from uncertainty to being informed’ and 4) ‘Relating & Alienating’. Individuals with very positive appraisals spoke most frequently of helpful relationships with both staff and other patients, and feeling cared for. They also spoke of having had previous admissions and the assessment process on entering the ward suggesting that these may be valuable experiences. Conversely, the group with very negative appraisals spoke of relationships that were alienating or where there was a perceived abuse of power. They described restrictions to their freedom, compared hospital to prison and generally had the view that hospital makes you worse.

**Conclusions:**

The experience of hospital within the first few days of admission determines whether an individual has a positive or negative experience of their inpatient care. Reducing the impact of uncertainty and promoting good relationships may help services to improve the initial experience of hospital admission and ultimately improve future outcomes for patients.

## Background

Last year, there were 101,589 admissions to psychiatric wards in England which represents 3.9 percent of those in contact with secondary mental health services [1]. The quality of inpatient admissions, traditionally measured against service-defined outcomes, has become increasingly focused on patient experience; in-line with NHS values [2]. It is especially important to consider the experiences of those admitted to mental health services, as they can be detained and treated against their will under the Mental Health Act [3]. Aside from the ethical imperative, a negative experience of hospitalisation has been linked to poorer engagement with community services and an increased likelihood of relapse (4).

Patient-reported satisfaction with psychiatric care has been consistently associated with more positive future outcomes such as fewer hospital admissions and symptoms following discharge [4–6]. This holds true when patient-reported satisfaction has been assessed within a few days of starting treatment, which has been termed the subjective initial response (SIR). This association was first found in psychopharmacological studies looking at satisfaction with antipsychotic medication [7, 8], but has since been observed for complex interventions such as day hospital and inpatient psychiatric treatment [9–14]. In studies of inpatient admission, a positive SIR has been associated with symptom improvement at three months [15], and a reduced likelihood of re-admission within the year [12, 13] even when controlling for symptom severity at the time of assessment and a number of other patient-level characteristics.

Whilst initial satisfaction with inpatient psychiatric treatment has been shown to predict clinical outcomes, it is unclear which patient-level and hospital-level factors are associated with these appraisals, although a recent systematic review highlighted that involuntary status, being on a closed ward and the experience of coercion were associated with dissatisfaction [16]. Even less is known about what underlies the SIR for inpatient admission, possibly due to the perceived difficulty in collecting data within the first few days of admission. A better understanding of what determines the SIR may help to identify potential aspects of the admission which could be improved, particularly for those who are more likely to experience their admission negatively.

The present study aims to explore the initial experience of hospitalisation. Given the predictive value of this time period, it is important to understand what contributes to these appraisals. Therefore, a second aim of the study was explore what might underlie both very positive and very negative appraisals of treatment and care in hospital.

## Methods

### Sample

We conducted short semi-structured interviews with patients who had recently been admitted to acute psychiatric wards across five hospitals in three Mental Health Trusts in London, England. The hospitals varied as to whether the wards were gender mixed or not. Participants were recruited over six months between March and September 2015.

Interviewees were recruited from a larger sample participating in COFI (Comparing Functional and Integrated systems of mental health care), an international multi-site study comparing continuity and specialisation systems of mental health care. The full COFI protocol is published elsewhere [17]. Briefly, individuals were eligible for the study if they had been admitted to an acute psychiatric inpatient ward and i) had a clinical diagnosis of psychotic disorder (F20-29), affective disorder (F30-39) or anxiety/somatisation disorder (F40-49) (International Classification of Diseases–ICD- 10) ii) were able to provide informed consent and iii) could speak and understand English. Ethical approval for the present study, including the consent procedures, was obtained from the National Research Ethics Committee based in Newcastle & North Tyneside (ref: 14/NE/1017).

Participants were recruited as close to their admission date as possible and within five days of that date. A purposive sampling frame aimed to maximise variation in the initial inpatient experience. As part of the COFI study, all participants completed a quantitative measure of patient satisfaction, the client assessment of treatment (CAT, [9]). The CAT is a brief seven item scale assessing different aspects of hospital treatment (e.g. psychiatrist, personal respect, medication etc.) with each rated on a scale from 0 to 10 where 10 indicates highest satisfaction.

To ensure that a wide range of views and experiences were represented, our sampling frame included satisfaction (satisfied vs. dissatisfied) as measured by the CAT, admission status (voluntary vs. involuntary) and whether or not an individual had previous experience of hospitalisation (first admission vs. previous admissions).

### Procedures

Initially, clinicians were approached by researchers and asked whether a patient would be well enough (e.g. likely to have capacity to consent) to take part in the research study. If it was deemed that the patient was likely to have capacity, the clinician then asked the patient for their assent to participate in the wider COFI study. Where assent was obtained, researchers were then introduced to patients, explained the study and obtained written informed consent. To assess capacity to consent, researchers checked the patient’s ability to use and understand the study information provided, made sure individuals understood the research and what would be involved and ensured the patient was able to communicate their decision. All researchers were trained in assessing capacity to consent. Following completion of the main COFI study i.e. the quantitative assessment, eligible participants were invited to take part in a short qualitative interview with the aim of understanding the experiences underlying their appraisals. Interviews took place there and then, on the hospital ward to which the participant had been admitted in a quiet and available room. The interviews were carried out by two female graduate research assistants (AC & EN) trained in qualitative interviewing and with experience conducting research assessments with this population.

The topic guide focused on the participant’s experience of their current admission and inpatient treatment, regardless of whether they had previously been admitted. To minimise interference with daily ward activities and research burden, the topic guide was designed to keep the interviews short (10–15 minutes). In the early stages of data collection, a small number of interviews (N = 4) were transcribed and assessed to inform adjustments to the topic guide. Notably, the modified topic guide included prompts to ground the interviews in what had happened during or since admission (e.g. “Take me through your first day”) and to steer participants away from only focusing on the circumstances which led up to the admission.

### Data analysis

All interviews were audio-recorded and transcribed verbatim by an independent transcription service. The transcripts were checked against the original data to ensure accuracy and for them to be pseudo-anonymised. This included removing reference to any personal information, including the names of any staff members of patients on the ward. Cleaned transcripts were uploaded into QRS International Nvivo version 11, which was used to manage and analyse the data.

In the first stage, thematic analysis was used to analyse the transcripts using the iterative steps outlined by Braun and Clark [18]: familiarisation with the data, axial coding, searching for emerging themes, reviewing and defining the themes. Analysis was inductive and based on the content of the transcripts rather than on any clear existing theory or hypothesis. Despite this inductive approach, the authors acknowledge that the analysis was likely to be shaped to some extent by their previous experience within a research and/or clinical capacity.

Three authors (AC, EN & VB) independently coded five interviews assessed to be representative of the data during the familiarisation stage. The researchers regularly met to discuss their initial codes, including any discrepancies, and how the codes may be linked in order to develop an initial coding framework. Following development of an initial framework, the three researchers coded another five interviews before the framework was further refined and agreed upon. This framework was then flexibly applied by one author (AC) to the rest of the interviews. During this phase, particular attention was paid to any new themes not captured in the initial coding framework. Throughout the process, the researchers regularly discussed changes to the framework and emerging themes with the wider team. In a final stage, another analyst (CF) independently applied the coding framework to 20% of the interviews to maximise the robustness of the framework.

Content analysis was used to achieve the second aim to the study and to understand which codes were linked to more positive and negative appraisals of hospitalisation. This was achieved by first calculating the upper and lower quartile of scores on the CAT in order to identify the participants with scores showing very high satisfaction or very low satisfaction. The frequency of the occurrence of each sub-theme within these two groups was calculated. The five sub-themes that occurred most frequently for each group are reported in order to explore what may underlie the experience of being very satisfied or very dissatisfied with the initial experience of inpatient care. Where two themes had the same frequency across participants, they were ordered based on the total number of occurrences within the dataset.

## Results

In total 61 interviews were conducted and with a median duration of 10:11 minutes (IQR 7:30 to 14:25). The majority of participants were male (67%), with over 50% of participants having a primary diagnosis of psychosis (54%). Full sociodemographic characteristics of the participants are reported in Table 1. The data was organised into four broad themes each with a number of sub-themes: 1) ‘Best place for me right now?’ 2) ‘Different from out in society’ 3) ‘Moving from uncertainty to being informed’ and 4) ‘Relating & Alienating.’ The full coding framework is shown in the S1 Appendix.

**Table 1:** Participant characteristics.

	Characteristic	Participants (N = 61)
Gender N (%)	Female	20 (32.8)
	Male	41 (67.2)
Admission status N (%)	Involuntary	23 (37.7)
	Voluntary	38 (62.3)
Admission history N (%)	First admission	26 (42.6)
	Previous admissions	35 (57.4)
Primary diagnosis N (%)	Psychosis (F2)	33 (54.1)
	Mood disorder (F3)	9 (14.8)
	Anxiety disorder (F4)	8 (13.1)
	Other	11 (18.0)
Hospital Trust N (%)	East London NHS Trust	24 (39.3)
	North East London NHS Trust	25 (41.0)
	Camden & Islington NHS Trust	12 (19.7)
Overall satisfaction score Median (IQR)		6.8 (4.1)

### 1. Best place for me right now?

This theme contains thoughts and feelings about being in hospital and whether the person believed they should be there. Opinions varied from viewing hospital as a space for respite to a space that made patients feel worse. Often informing these views were respondents’ understanding of how the admission decision was made, which ranged from having made the decision themselves to feeling powerless in that process. In addition to these contrasting views, others were more ambivalent about their admission. In these cases, it was often less clear who the decision-maker was, for example if family members were involved.

#### 1.1 Feeling safe

Some participants expressed a feeling of safety, which was in contrast to how they felt before hospitalisation, where they felt out of control or were experiencing symptoms (e.g. hearing voices)

*“I’m in a better place where [than] I would be if I was on the streets- you know ‘cos things would have spiralled out of control”* (103589)

Many of these people had admitted themselves or agreed to the admission, having unsuccessfully tried different treatment options. However, this was not always the case; some involuntary patients also found refuge in hospital and were grateful that someone had made the decision for them when they were too unwell to decide for themselves.

Reassurance from staff contributed to feelings of safety or security, with hospital offering a safety net. For example, a patient who had previously been admitted to the same ward described feeling comforted by the familiarity of staff, in the context of being quite socially isolated in the community.

*“[It’s a] nice feeling*. *It’s almost like, like hmm, having time off work, and coming back into the workplace and everyone’s happy to see you.”(102554)*

#### 1.2 Hospital makes you worse

At the other end of the spectrum hospital was viewed as a negative environment, one that can result in feeling worse. Some participants spoke about how the restriction on freedom made them feel restless, or as one patient put it, ‘crazy’. Others spoke about it as an environment that was more isolating than being at home or one which exacerbated feelings of hopelessness.

*“I thought [in-breath] locking someone up somewhere*, *in one place*, *isn’t gonna make them better*, *it’s gonna make them worse; it’s gonna make them crazy*.*”* (1011086)

Some participants questioned the suitability of psychiatric care more generally, questioning whether treatment predominantly focused on medication, was effective and wondered whether it should include more talking therapies. Others questioned the power held by mental health care professionals and these institutions‘.

*“The scope for abuse is massive because it is a subjective decision (…)*, *the detention, and the continuation of the detention is made by one single psychiatrist” (102474)*

#### 1.3 Acceptance

In other cases individuals had more nuanced views about their admission. This included patients that were displeased about being admitted, but had resigned themselves to it. These were often involuntary patients or patients who only agreed to the admission or treatment due to threat of coercive measures (i.e. informal coercion), such as being forcibly admitted if they did not. For others, this view was based on previous admissions, where they had learnt that this was the best option. Generally, there was a sense that they just had to get on with it and wait until their discharge.

*“I know if I refuse, they could lock you up in here for months on end and pin you down with a needle and all that*, *I don’t think I needed their chemicals but, I suppose now I swallow the tablet and bite me tongue” (102501)*

Another group also accepted that they had to get on with being in hospital because whilst not ideal, it was for their own good. In these cases, hospital was seen as having both costs and benefits; and this acceptance was sometimes presented as a process occurring over time. The admission in general was compared to a learning experience in that it was challenging and unfamiliar but provided a space to think about the changes they needed to make for recovery and to stay well in the future.

*“I’ve had time to think over the*, *you know the past few days [in-breath] and I’ve realised this is good for me because [in-breath] um*, *it gives me time to be alone–on my own–away from my family and my friends*, *[in-breath] and it gives me time to think about what…what I can do to change myself and not come back here*.*”* (101186)

### 2. Different from out in society

For many, aspects of the ward were experienced as different or cut-off from the outside world. Like other institutions, psychiatric wards are governed by their own set of rules and routines apparent even within the first few days of admission. What came across strongly was a lack of occupation or boredom experienced on the ward, often in stark contrast to their lives outside of hospital.

#### 2.1 Institutions

Many people referred to hospital being like a prison or used prison-like language to describe aspects of the ward (e.g. referring to other patients as ‘inmates’ or anticipating a ‘release date.’) The design of the ward with separate rooms radiating off a corridor and restrictions on access to different parts of the ward, were other points of comparison.

*“I’ve been locked up in here with no sentence, with no trial, with anything*. *I’ve just been taken from my home and locked up” (103871)*

Prison was not the only institution hospitals were compared to with some individuals comparing it to a hotel, a place where you are cared for, and another comparing it to a school; as a place for personal growth with set activities and mealtimes. Throughout there was a sense that the ward was cut-off from the outside world, which came through in descriptions of feeling lonely or missing family members.

*“I don’t have any contact with the rest of the world you know, with my fiancé and kids… that’s been really difficult” (102836)*.

#### 2.2 Rules & restrictions

People spoke about the restrictions inherent in an institution, which were often aligned with feeling closed-in or aggravated. Following rules, especially respecting smoking restrictions, was frustrating. One patient noted that most people were smokers and that this felt especially restrictive given the lack of other activities provided on the ward.

“*It’s quite hard to think oh shit*, *8 o’clock I can’t smoke no more*. *That*, *that’s kind of put me on edge*, *as it were*” (1011197)

Depending on the legal status of the individual, another restriction was the lack of freedom to leave the ward, go to the shops or to get fresh air at will. This led some to feel claustrophobic or as though they had excess energy to expend.

*“When I got told that I can have two hours out a day escorted that obviously helped*. *[…] but that two hours out of a day when you’re an active person is not much”* (103509)

People also talked about restrictions to their privacy; which included being searched during admission, having personal items removed, or the experience of being under observation–which often felt suffocating.

*“I promised them that I’m not gonna hurt myself here*, *so I don’t know why they can’t just give me a bit of space.” (102851)*.

On the other hand, the positive side of these rules and restrictions included the structure and guidance around how to behave that they provided.

#### 2.3 Gateway between hospital and the outside world

Meetings with doctors, or ward rounds, could be perceived as gateways into and out of hospital contributing to the notion that it is cut-off from the rest of society. On entering the ward, patients are assessed, and this process often felt long and without urgency.

*“If I haven’t seen a doctor yet, how is the emergency over*? *But yet they may keep you waiting for two hours, like the emergency is over once you get into the building–and that’s not good” (101689)*

The process of discharge could also feel obstructed. The ward rounds were often experienced as pressured and intimidating because they were a rare opportunity for important questions to be answered and decisions to be made, including discharge decisions. Adding to the pressure of ward rounds was the fact that many patients were assessed by many different health professionals at once.

#### 2.4 Boredom

Aside from mealtimes and meetings with doctors, there was not much to punctuate time on the ward and most spoke about boredom at some point. It was particularly pronounced at the weekend when there were fewer appointments or ward activities. The lack of occupation was difficult especially when looking for a distraction from symptoms or rumination.

“*I don’t like to sit doing nothing because that’s when my*, *my mind just starts going into overtime”* (102503).

Where groups or activities were offered, they could be experienced as childish or inappropriate. Similarly when entertainment, such as table tennis or books were provided there often was not enough to go round or the materials were broken.

“The *activities are very um*, *childish maybe if there was a little bit more activities during the day for actual adults rather than*, *you know colouring in by numbers and that type of stuff*” (1011051)

### 3. Moving from uncertainty to being informed

This overarching theme captures the two contrasting experiences of not knowing what is going on through to feeling well informed. Such experiences start from before the admission when expectations are formed, when patients first experienced the ward and throughout their first few days in hospital. Experiences of uncertainty or a lack of information were described alongside feeling anxious or distressed. There was also a sense that as the days went on, through a process of information gathering and becoming used to the environment, things got better and this anxiety reduced.

#### 3.1 Not knowing what to expect

People for whom it was their first admission spoke about not knowing what to expect and feeling daunted. Some went further explaining they had never formed expectations about what a psychiatric ward would be like having never expected that they would end up there.

*“I don’t have any experience to go before any mental hospital, I don’t go before so I don’t know what this is compared. If I go next time*, *then I’ll compare […] In my life I didn’t, I never think ‘How is mental hospital?’”(101829)*

Those that had some prior expectations drew on the experiences of friends or family as well as on media portrayals. These descriptions often presented hospital as a place that was quite strange or mysterious.

*“I expected it to be like something off…- have you ever seen the film Charles Bronson? […] there’s a mental ward on there. Where they play a xylophone in the middle of the room with one […]–you know with a big speaker [I: Alright, right]*, *with one track playing continuously.” (103811)*

#### 3.2 Confusion and chaos

Whilst a sense of confusion runs through the theme, this was especially prevalent during the admission. When asked about the admission process, some patients noted that their memories were hazy, and drew attention to the fact that they were most likely too unwell to take on much information at the time.

*“To be fair I was so like, still incapacitated*, *I didn’t really know what was going on.” (102882)*

Along with feeling confused, many remembered feeling frightened or overwhelmed on first entering the ward. The direction of this relationship was unclear—in some cases these feelings contributed to the confusion whilst in others, the uncertainty added to them.

*“R: I was frightened. (I: Okay*, *in what way?) R: I didn’t know what was gonna happen.” (101929)*

Following the admission itself, the ward could be experienced as a chaotic place, with other patients positioned as noisy and agitated and with too few staff available to contain the atmosphere. Some spoke about how staff are overstretched, because of increasing demand on mental health services and in some cases this made participants worry or feel somewhat unsafe.

*“if something kicks off then you’ve got three or four of ‘em that are concentrating on the two people that are going [0*.*2] ballistic or whatever” (102778)*

#### 3.3 Communication and information

A lack of information or clear communication experienced during the admission and on the ward contributed to this sense of uncertainty.

*“it was only today that I’ve…sort of got, I mean, got to know a bit about the place: you know I’ve got my Welcome Pack, I’ve got the rules, I’ve got the…what’s going on during the day*. *Um, … so yeah, it was a bit sort of um, in at the deep end” (102478)*

Other times, stress was related to a lack of communication between staff and patients or their family members in relation to treatment. Some patients expressed that they hadn’t been given feedback on test results, given information they had requested or updated with changes to their care plan. This was distressing and gave the impression that staff did not care.

*“It’s hard enough as it is so it would have been nice for a little bit more of a sit down ‘do you know where you are?, ‘do you know what’s going to happen to you?*’… *I didn’t know how long I was going to be here for—I still don’t know how long I am going to be here for” (102916)*

However, some felt that they had been given sufficient information, updates or reassurance from staff, which in turn was generally associated with positive feelings.

*“The Doctor talked to me, and showed me the ward, they gave me belief*, *they gave me confidence that everything would be fine …” (101829)*

#### 3.4 You get used to it, it gets better

Perhaps linked to this dichotomy between uncertainty and having information is the notion that things get better as time goes on. Some people made reference to an adjustment period where they had to adapt to the routine of the ward or to sharing the space with other people.

*“All of it was a surprise, it was horrifying in some ways, my anxiety has just been through the roof … but then again now I’ve been here*, *I’ve got used to the system” (102916)*

When asked why things got better, people spoke about getting to know the place, gaining a better sense of why they were in hospital and how long they were going to be there. Even when they didn’t know how long they were going to stay, this was more easily tolerated as they became used to the ward. With time people also learnt to accept some of the restrictions on the ward, such as prescribed smoking or leave times, which helped to improve the experience.

In line with this idea, those with previous experience of hospitalisation and/or well-formed expectations, were less likely to report this initial anxiety.

*“What was your first impression when you arrived here in this ward? (laughs) Well I am used to it! When I arrived, I knew I was gonna get to see the doctor […] they ‘re gonna keep me in, they’re gonna review my medication*, *I knew all these things”*

### 4. Relating & alienating

Relationships on the ward tended to fall into two camps, those that were therapeutic and those that were alienating. Therapeutic interactions tended to occur when respondents felt others were able to relate or understand them. This was in stark contrast to respondents that positioned themselves as different to other patients and therefore kept to themselves. Similarly, some respondents felt alienated by staff. This was evidenced by the experience of staff keeping themselves separate or experiences which were perceived as an abuse of power by patients.

#### 4.1 Social interaction as therapeutic

Being in hospital entails being around others and the company contrasted to the isolation experienced at home. Even without social interaction, the very act of being around other people was appreciated.

*“it is a marked change from the isolation and so the company is very therapeutic*” *(102474)*

Some patients felt a sense of camaraderie towards other patients. Having a similar shared experience of hospital, mental illness or associated stigma meant that they were able to relate to each other and not feel judged–this was experienced as supportive.

*“We’re not crazy people we’re just people that have issues*” *(102882)*

Lots of positive adjectives were used to describe staff such as ‘friendly’, ‘efficient’, ‘patient’. Whilst many did not elaborate, a few patients went further and explained that these qualities made them feel cared for. Patients recounted specific instances when staff went “above and beyond” but also felt comforted when someone noticed they were alone and just approached them for a simple chat.

*“Staff have been wonderful*, *they really are, […] there’s nothing, they can’t do for you. It’s like, I don’t drink juice, and at lunch time […] they only put out juice, but they’ll always make me a cup of tea” (101918)*

#### 4.2 Individuals feeling they are different to other patients

One group of respondents viewed themselves as different from other patients, often viewing themselves in a more positive light. The comparisons were both, explicit or subtle references to other patients being more unwell, vulnerable, noisy or unpredictable.

*“I feel like I’m kind of like with a broken bone in a burn ward–if that makes sense […]as in like there’s people who are very mentally ill, and I’m*, *I’m quite […] well ‘stable’”(103754)*

Feeling different from other patients could act as a barrier to social interaction. Some patients preferred to spend time alone in their rooms and others would avoid social interaction because they felt that other patients did not respect their personal boundaries.

*“I try and keep myself out the way of the other patients–the other patients wind me up*” *(102764)*

This othering led to suggestions that patients should be on different wards according to the type or severity of their symptoms. Some even suggested that patients be placed on separate wards according to age group explaining that young people were noisy and that this was not conducive to recovery.

#### 4.3 Them & Us

This sub-theme captured the feeling that staff positioned themselves as superior to patients and kept them at a distance. Participants spoke about nurses being present in body but difficult to access in that they spent most of their time in the ‘nurses’ station’. These offices are often centrally located with big windows looking out onto the ward and a locked door. Having to knock on the door to attention made some patients feel uncomfortable and as though they were a burden. Others spoke about knocking on the door and being ignored which made them feel alienated.

“*you know they go in their office*, *you have to knock on their door*, *like [you’re] bothering ‘em*. *They should be out more asking “are you okay*, *what can I do for you*, *is there anything you need help with” rather than sitting*, *sort of laughing and drinking tea*.*” (102764)*

As well as a physical separation, nurses and doctors were experienced as distant in their interactions. This was evidenced by patients feeling as though they weren’t being treated as individuals but that staff ‘tar everyone with the same brush’ and that assumptions were being made.

*“Well they were like um, making statements, instead of asking questions first*, *and then they started asking me questions, which they already knew the answer to anyway” (102577)*

Othering could work in the opposite direction whereby patients viewed staff as threatening or untrustworthy. Some even felt that these qualities were inherent to mental health professionals. These views could be due to explicit or implicit threat of coercive measures as highlighted in this example:

*“if I don’t comply or if I [in-breath] don’t want to take my medication, or disagree with something or get upset about something the first thing they are gonna do is er*, *[…] do what, do what they did last time.” (102464)*

Some also kept themselves away from staff for fear that they would be judged or that what they said could be used against them.

*“As soon as I get angry they are going to say that something wrong with me*. *Even if I am just very passionate about a subject or something that is making me angry, they will just say that it is part of my mental health” (102920)*.

### Themes common to positive and negative appraisals of care

The content analysis indicated the top five themes most prevalent for individuals scoring in the top and bottom quartiles on the CAT, representing either a positive or a negative initial appraisal of care respectively. Although there was overlap with both positive and negative themes apparent in the experiences of individuals, as shown in Table 2., the five most prevalent themes included in positive appraisals of care, were distinct from the five most prevalent themes included in negative appraisal.

**Table 2:** Themes most common to positive and negative appraisals of care.

Positive appraisals		Negative appraisals	
Theme	Number of Sources	Theme	Number of Sources
Positive qualities of staff	9	Lack of access	8
Feeling cared for	8	Perceived abuse of power	7
Previous experience of hospitalisation	7	Individuals feeling they are different from other patients	7
Patients supporting each other	6	Hospital as a prison	7
Interviewed and assessed as part of the admission	6	Hospital makes you worse	6

## Discussion

Four broad themes, developed from the data, captured the initial subjective experience of being admitted to an inpatient psychiatric ward. The first three themes are conceptually linked in that they describe feelings of uncertainty and confusion. Within these themes, one speaks to the variety of, and often mixed, views that individuals can simultaneously hold about their admission and how these are influenced by the sense of control that one has over the decision to be admitted (Best place for me right now?). A second notes how different the ward environment is to the outside world and how unfamiliar this is to some, with comparisons made between hospital and other institutions (Different from out in society). The third theme contains more explicit experiences of distress often occurring during periods of uncertainty, and described how this is on a continuum with becoming fully informed (Moving from uncertainty to being informed). The final theme, which sits slightly aside from the others although prominent throughout the analysis, focuses on relationships with both staff and other patients, describing how some can be conducive to recovery whilst others can feel alienating or detrimental to their wellbeing (Relating & Alienating).

Although there was a large amount of overlap between themes apparent for individuals with either overall positive or negative appraisals of care (as measured on the CAT), the content analysis indicated distinct themes common to these experiences. For individuals with an overall positive appraisal of their admission, hospital was viewed as a supportive environment, where individuals recognised the positive qualities of staff members and of other patients, and felt genuinely cared for. Interestingly, many of these individuals with positive appraisals described having had previous hospitalisations, an experience which may have reduced the uncertainty and confusion commonly described throughout the interviews. This group also spoke frequently of their initial assessment on entering the ward suggesting that this was a process that was understood and felt collaborative.

In contrast, interviews of those with very negative appraisals were characterised by restrictions on the individual and a perceived abuse of power that often linked to and contributed to the description of the hospital as an institution. Rather than seeing patients as supportive, individuals with a more negative appraisal tended to see themselves as different from others, contributing to the notion that hospital was not the place for them and could in fact make them worse.

### Strengths and limitations

This study is to our knowledge the first to use patient interviews to explore the experience of being admitted to a psychiatric inpatient ward within the first few days of admission. In the present study took place on the ward itself, usually within the first two days, and always within the first five days of the admission. This is in contrast to previous studies (e.g. [19, 20]), which have assessed the experience of inpatient care retrospectively, often within the community, which may introduce interference from recall bias. Similarly, retrospective interviews may include interpretations and meanings attributed to the experience itself, which develop over time or following discharge.

Another strength of this study was the heterogeneity of the included sample. Participants were drawn from a larger study of inpatient care (i.e. the COFI study [17]) the inclusion criteria for which was intentionally broad. Additionally, within the sampling frame, we aimed to interview individuals that are both satisfied and dissatisfied with their admission, with both voluntary and involuntary status, and both those with either a first or repeated admission to hospital to gather a range of initial experiences.

A final strength of the paper was the use of both qualitative and quantitative data to explore initial satisfaction with services. This is especially relevant given that recent research has highlighted the difficulty in being able to attach meaning to survey data of patient experience, where negative experiences assessed with interviews can underlie ‘good’ ratings [21].

Despite these strengths, there were a number of limitations to the study. Firstly, individuals were included from only five hospitals all within the London area. Although the sampling frame aimed to maximise variation in patient characteristics, including individuals from different urban, semi-urban and rural locations may have resulted in different experiences of hospital care. Secondly, whilst, interviewers tried to reduce demand characteristics by emphasising that they were independent from the clinical team, it is still possible that respondents may have deliberately concealed certain views. Similarly, some participants may not have felt comfortable or felt they had the opportunity to go into too much depth about their experiences due to the brevity of the interviews. Whilst we did not terminate interviews mid-flow, we explained that they would be relatively short. This ensured that we did not systematically exclude a certain group of patients, especially those with difficulty concentrating over longer periods of time. Additionally, interviewers had spent time with the participant, completing the informed consent process and the quantitative assessment, thus enabling them to build a rapport with the patient. Finally, although interviewers were trained in taking informed consent and ensured individuals had capacity to take part, participants were sometimes unwell. Thus, although a main strength of the study, interviewing within the first few days of admission did present some challenges. These included working with clinicians to gain assent, ensuring the participant retained capacity throughout and interpreting the data whilst being mindful of content that may be thought disordered.

### Comparisons with the literature

A systematic search and thematic synthesis of qualitative research on patients’ overall experiences of psychiatric inpatient care [22] identified three superordinate themes. These were i) collaborative and inclusive care (control and involvement in one’s care plan, medical versus psychosocial approaches & transition of leaving hospital), ii) positive relationships (relationships with staff, interactions with other patients, family & friends) and iii) safe and therapeutic environments (hospital as a haven, physical aspects of a hospital, daily life & safety inside hospital). Although many of the themes were common to the present study–most notably the overarching theme on relationships, only three out of eleven studies included in the review were conducted whilst the participant was still an inpatient [23–25]. Instead, most focused on their retrospective appraisal of care following discharge [26] or more generally asked about previous experiences of hospital admission [27]. Additionally, user-led research, which including interviewing patients in the community following admission, also highlighted the importance of relationships. As with the present study, Gilburt and colleagues indicated that forming a positive relationship with staff, which included effective communication and was experienced in the absence of any perceived coercion, was the cornerstone to developing trust. This in turn was linked to viewing hospital as a safe and supportive environment, a theme echoed in the positive appraisals included in the present study[28].

Although the above research adds to and provides valuable insights into the experience of care, previous research has indicated that an individual’s appraisal may differ throughout their hospital stay [29]. In particular, appraisals may become more positive as people reflect back on their experience, especially following discharge. Indeed, within the present study we found that participants reported a period of adjustment following admission, with many people becoming resigned to staying in hospital, or learning to make the best of it. This was the case for both voluntary and involuntary patients, and was most notably captured in the theme “moving from uncertainty to information”.

Although not present in all quantitative studies assessing the association between patient characteristics and satisfaction, a number of studies have found that individuals with a diagnosis of schizophrenia and related disorders are less satisfied with services [30–32]. Specifically, high levels of positive symptoms or hypomania were associated with lower treatment satisfaction within the first few days of admission compared to patients with fewer symptoms [31]. This is interesting given the present finding that uncertainty pervades many aspects of the first few days of admission and that this may be compounded by the experience of illness.

At the level of services involuntary admission, being on a closed ward and subjective experiences of coercive measures are most consistently associated with lower levels of satisfaction [16]. Indeed in the present study the most frequent themes for patients that were highly dissatisfied were ‘lack of access’, especially with regard to leaving the ward, and a perceived abuse of power, which included the experience of coercion, both of which have previously been noted in other qualitative studies [33, 34]. However in all of these studies the focus is on the patient perception and does not include the clinician viewpoint who may regard this as necessity.

In contrast, good staff-patient relationships seem to be an important factor in informing satisfaction [16, 28]. Similarly, a large survey assessing factors associated with satisfaction across 11 countries found low staff to patient ratio to be the only service level characteristic associated with poor satisfaction [30]. Taken together these reflect the finding that among those most satisfied with their admission, ‘positive qualities of staff’ and ‘feeling cared for’ were the most important themes.

Finally, service organisation has also been shown to have an impact on levels of satisfaction. For example, in the larger study from which this sample was drawn, it was demonstrated that patients had higher levels of satisfaction if admitted to a hospital utilising sectorised care, whereby patients see the same psychiatrist across inpatient and outpatient settings, compared to functional care, where patients see different psychiatrists across settings [35]. Again this highlights the role that staff-patient relationships may play in satisfaction.

### Implications

Aside from the difficulty in comparing across studies, quantitative measures of patient satisfaction may not provide the rich understanding of hospitalisation needed in order to identify areas for intervention. In the present study, we used quantitative CAT ratings to sample individuals with negative and positive appraisals, using the quartiles as a way to classify people as either highly positive or highly negative. This helped to overcome another limitation with quantitative measures of satisfaction, which is the propensity for patients to quantitatively rate experiences more positively compared to their qualitative evaluation [21]. Within our analysis, we were able to indicate clear and distinct themes and sub-themes associated with both positive and negative appraisals–regardless of the overall higher rating of satisfaction often seen when using quantitative measures.

As initial satisfaction has been linked to more positive clinical outcomes, including treatment outcomes within the community [13], understanding what constitutes a positive or negative appraisal of care may help services target certain experiences. For example, our analysis suggests that emphasising and increasing the experiences linked with positive appraisals such as providing information during and after the admission, ensuring where possible that individuals are included in decisions and fostering a caring environment by reducing the physical and mental separation of staff from patients within the ward may increase positive appraisal of care. Furthermore, the analysis could suggest the need to directly reduce the impact of practices such as informal coercion and address the perceived lack of power, both of which were consistently linked with lower satisfaction rates.

Understanding what drives positive and negative appraisals through mixed-methods investigations, may be particularly crucial for involuntary patients, where research has shown initial satisfaction to be the only predictor of clinical outcomes one year following an involuntary admission [14]. Within the present study, we included both voluntary and involuntary patients. Importantly the analysis indicated that not all involuntary patients viewed hospital as a negative experience, with many seeing the positives of an admission and learning to deal with the hospital environment even within a few days of admission. However, lack of access and information were still two main areas of concern, especially for involuntary patients.

### Conclusions

Initial satisfaction with inpatient care is an important outcome in its own right and one linked to better clinical outcomes in both the short and long term. Understanding the experiences contributing to both negative and positive appraisals of care may help services to improve satisfaction for patients. Identifying the elements linked to appraisals, such as providing information or reducing feelings of coercion provides areas that interventions could target if wanting to improve the initial experience and hence potentially improve clinical outcomes in the long-term.

## Supporting information

S1 Appendix Full coding framework. (DOCX)

S2 Appendix Topic guide. (DOCX)
