# Supplementary material for: Exploring the initial experience of hospitalisation to an acute psychiatric ward
Source: PLoS One. 2018 Sep 4;13(9):e0203457. doi: 10.1371/journal.pone.0203457 (PMC6122813; doi:10.1371/journal.pone.0203457)
Supplement: S1 Appendix — (DOCX) [file pone.0203457.s001.docx]

**S1 Appendix. Full coding framework**

|  | | | | | ***Level 2 themes*** | ***Level 3 themes*** |
| --- | --- | --- | --- | --- | --- | --- |
| 1. **Best place for me right now?** | | | | | | |
|  | - 1. **Feeling safe** | | | | | |
|  |  | |  | | **Hospital environment as calming or pleasant** | Familiar, contained |
|  |  | |  | |  | Nice and friendly atmosphere |
|  |  | |  | | **Feeling safe** | Relief at having somewhere to stay |
|  |  | |  | | **Happy or grateful for care received** | Getting the support that I need |
|  |  | |  | |  | Grateful (for NHS) |
|  |  | |  | | **My decision to come to hospital** | Have tried other options, last resort |
|  |  | |  | |  | Wanting to help/to be admitted |
|  | - 1. **Hospital makes you worse** | | | | | |
|  |  |  | | | **Hospital makes you worse** | Feeling unsafe |
|  |  |  | | | **Questioning the validity of psychiatry** | Should be better access to talking therapy |
|  |  |  | | |  | They shouldn’t use restraint |
|  |  |  | | |  | Not thinking you need medication |
|  |  |  | | | **Disagreeing with admission** | Involuntary admission |
|  |  |  | | |  | I shouldn’t be in a psychiatric hospital |
|  |  |  | | | **Anticipating going home** |  |
|  | - 1. **Acceptance** | | | | | |
|  |  | | **Resignation** | | **Making the best out of a bad situation** | Bearing it |
|  |  | |  | | **Lack of control, feeling disempowered** | Complying |
|  |  | |  | |  | Small acts of defiance |
|  |  | | **For my own good** | | **For my own good** | Blessing and a curse |
|  |  | |  | | **Space to think** | Realising I need to make changes |
|  |  | |  | |  | To slow down thoughts |
|  |  | | **Not entirely my decision** | | **Admission was a shared decision** | Planned admission |
|  |  | |  | | **Informal coercion on admission** | Putting words into my mouth |
|  |  | |  | | **Carers involved in the decision** | Hostel staff |
| 1. **Different from out in society** | | | | | | |
|  | - 1. **Comparisons with other institutions** | | | | | |
|  |  | | **Institutions** | | **Hospital as a prison** |  |
|  |  | |  | | **Hospital as a school** |  |
|  |  | |  | | **Hospital as a hotel** |  |
|  |  | |  | | **Full of ‘nutters’** |  |
|  |  | | **Missing family** | | **Missing family** | I wish they would visit |
|  | - 1. **Rules & restrictions** | | | | | |
|  |  |  | | | **Having to follow rules** | Being told what to do |
|  |  |  | | |  | Not allowed to smoke |
|  |  |  | | |  | Staff rigidly enforce rules |
|  |  |  | | | **Lack of access** | Access to outside |
|  |  |  | | |  | Doors are locked |
|  |  |  | | |  | Feeling house-trapped |
|  |  |  | | | **Lack of privacy** | Personal items being removed |
|  |  |  | | |  | Searched during the admission |
|  |  |  | | | **Rules as positive** | Provides structure |
|  |  |  | | |  | Learning to live independently |
|  | - 1. **Gateways between hospital and the outside world** | | | | | |
|  |  | | | **Assessment** | **Interviewed and assessed at admission** | Physical examinations and tests |
|  |  | | | **Waiting times at admission** | **Long waiting times at admission** | Lack of available beds |
|  |  | | |  |  | Waiting to be assessed |
|  |  | | |  | **Timely intervention** |  |
|  |  | | | **Ward rounds** | **Long wait to see a psychiatrist** | Especially at weekends |
|  |  | | |  |  | Appointments cancelled |
|  |  | | |  | **Ward round as intimidating or pressured** | Fearing the psychiatrist |
|  |  | | |  |  | Having to prove I am sane |
|  |  | | |  |  | Lots of people, intimidating |
|  | - 1. **Boredom** | | | | | |
|  |  | | | **Feeling bored** | **Feeling bored** | Need things to distract you |
|  |  | | |  | **Lack of appropriate activities** | Less activities at the weekend |
|  |  | | | **Structured routine** | **Routine is positive** |  |
| 1. **Moving from uncertainty to being informed** | | | | | | |
|  | - 1. **Not knowing what to expect** | | | | | |
|  |  |  | | | **First admission** | Never expected to be here |
|  |  |  | | | **Some prior beliefs or expectations** | Family members have been unwell |
|  |  |  | | |  | Societal or media portrayal |
|  |  |  | | |  | Personal beliefs |
|  | - 1. **Confusion and chaos** | | | | | |
|  |  | | | **During the admission** | **All a bit hazy** | Being too unwell to take in information |
|  |  | | |  | **Feeling frightened or overwhelmed** | Having questions, doubts |
|  |  | | | **Since the admission** | **Hospital environment as stressful, hectic** | No protocol |
|  |  | | |  |  | Noisy |
|  |  | | | **The ward is understaffed** | **The ward is understaffed** | Need more staff to patients |
|  |  | | |  |  | Need more staff at weekends, chaotic |
|  |  | | |  |  | Staff can’t do their jobs properly |
|  | - 1. **Uncertainty vs. having a plan in hospital** | | | | | |
|  |  | | | **During the admission** | **Lack of information or manual** | No introduction to staff - who is who |
|  |  | | |  | **Being informed during the admission** | Already know about process or routines |
|  |  | | |  |  | Given an introduction to the ward |
|  |  | | |  |  | Being reassured by staff |
|  |  | | | **Since the admission** | **Not understanding the ‘rules’** |  |
|  |  | | |  | **Lack of communication** | Promised things that don’t happen |
|  |  | | |  |  | No communication with carers |
|  |  | | |  |  | Not told about my rights |
|  |  | | |  |  | Not being updated with care plan |
|  |  | | |  | **Having information, having a plan** | Being given information |
|  |  | | |  |  | Staff explain things to you |
|  |  | | |  |  | Developing or putting a plan in place |
|  | - 1. **You get used to it, it gets better** | | | | | |
|  |  | | |  | **Hard to adjust, a bit of a shock** |  |
|  |  | | |  | **Initial impression has changed for the good** | Because of receiving information |
|  |  | | |  |  | Becoming involved in raising issues |
|  |  | | |  | **Previous experience of hospitalisation** |  |
| **4. Relating and alienating** | | | | | | |
|  | - 1. **Social interaction as therapeutic** | | | | | |
|  |  | | | **With patients** | **Patients supporting each other** | Being able to relate to other patients |
|  |  | | |  | **Wanting social interactions** | Having people to talk to |
|  |  | | |  |  | Interaction as therapeutic |
|  |  | | | **With staff** | **Positive qualities of staff** | Nice, friendly |
|  |  | | |  |  | Feeling respected |
|  |  | | |  | **Feeling cared for** | Efficient staff |
|  |  | | |  |  | Psychiatrist interested in me, listened |
|  |  | | |  |  | Trusting the psychiatrist’s expertise |
|  |  | | |  | **Staff as people to talk to** | Talking about everyday things |
|  | - 1. **Individuals feeling they are different to other patients** | | | | | |
|  |  |  | | | **Feeling different to other patients** | Nothing in common |
|  |  |  | | |  | Being frightened by other patients |
|  |  |  | | |  | They are more vulnerable |
|  |  |  | | |  | Other patients are annoying |
|  |  |  | | |  | Difficulties interacting with other patients |
|  |  |  | | |  | They are too unwell |
|  |  |  | | | **Not wanting to interact with other patients** | Not being able to get away from other patients |
|  |  |  | | |  | Wanting to be alone |
|  |  |  | | | **Patients should be separated based on …** | Severity of illness |
|  |  |  | | |  | Type of disorder |
|  |  |  | | |  | Age |
|  | - 1. **Them & Us** | | | | | |
|  |  | | | **Them & Us** | **Staff are difficult to access** | Psychiatrist should be more accessible |
|  |  | | |  | **Feeling ignored by staff** | Feeling like a burden or bothersome |
|  |  | | |  |  | Don’t get too involved, emotionally guarded |
|  |  | | |  | **Staff treating everyone the same** | Don’t know enough about you and your history |
|  |  | | |  |  | They make assumptions |
|  |  | | |  |  | Having to repeat your story |
|  |  | | | **Power imbalance** | **Perceived abuse of power** | Coercive measures |
|  |  | | |  |  | Informal coercion |
|  |  | | |  | **Don’t trust the staff** | Don’t trust the psychiatrist |
|  |  | | |  | **Negative attitude** | Rough |
|  |  | | |  |  | Egotistical or proud |
|  |  | | |  |  | Staff telling you off |
|  |  | | |  |  | Issues with specific staff |
