# Supplementary material for: Exploring the initial experience of hospitalisation to an acute psychiatric ward
Source: PLoS One. 2018 Sep 4;13(9):e0203457. doi: 10.1371/journal.pone.0203457 (PMC6122813; doi:10.1371/journal.pone.0203457)
Supplement: S2 Appendix — (DOCX) [file pone.0203457.s002.docx]

| Unit for Social and Community PsychiatryBarts & the London School of MedicineQueen Mary University of London |
| --- |

**TOPIC GUIDE**

Title of Project: **Comparing integrated and functional systems of mental health care (COFI) Additional qualitative interview**

**Before**

1. Can you tell me what was going on for you around the time you were admitted to hospital?

- Why were you admitted to hospital?
- Was it your decision to be admitted?
- Whose decision was it for you to be admitted?
- Did someone bring you here?

**Arrival**

1. What was the admission process?

- What happened when you arrived?
- What time of day was it when you arrived?
- What were your first impressions?
- How were you introduced to the ward? Did anyone show you around?
- Who did you see when you first came here?
- Have you seen a Doctor? How long did it take?
- Was anything surprising or unexpected?

**Since**

1. What has happened since you’ve been here? (take me through your first day)

- Are there things that you like or dislike?
- Is there anything that could be improved could be improved?
- How has it been with staff?
- What about with other patients?

1. Has anything changed your view/impression of the ward?
2. Has anything differed from your expectations?

**Environment**

We have been talking about your experience of care so far.

Now we’ll move to a slightly different topic.

We will talk about environment.

1. Please tell me what do you like about the hospital environment?

PROMPTS

- Here you can think about the way rooms are designed, colours of walls on wards, views from windows, plants, light, shared spaces…

- Where are your favourite places to go in the hospital and why?

- What places are least favourite and why?

2. Please tell me how do you find green areas, such as parks and gardens around the hospital building?

PROMPTS

- Are there enough green areas outside the hospital and are they well-kept?

- How do you access outside areas, for example to enjoy fresh air and sun?

- Are you able to enjoy views of nature from the ward?
